# The Inovirus Pf4 Triggers Antiviral Responses and Disrupts the Proliferation of Airway Basal Epithelial Cells

**DOI:** 10.3390/v16010165

**Published:** 2024-01-22

**Authors:** Medeea C. Popescu, Naomi L. Haddock, Elizabeth B. Burgener, Laura S. Rojas-Hernandez, Gernot Kaber, Aviv Hargil, Paul L. Bollyky, Carlos E. Milla

**Affiliations:** 1 Department of Infectious Diseases, Stanford University, Stanford, CA 94305, USApbollyky@stanford.edu (P.L.B.); 2 Immunology Program, Stanford University, Stanford, CA 94305, USA; 3 Center for Excellence in Pulmonary Biology, Stanford University School of Medicine, Stanford, CA 94305, USA

**Keywords:** inovirus, Pf4, *Pseudomonas*, cystic fibrosis, basal epithelial cells

## Abstract

Background: The inovirus Pf4 is a lysogenic bacteriophage of *Pseudomonas aeruginosa* (*Pa*). People with Cystic Fibrosis (pwCF) experience chronic airway infection with *Pa* and a significant proportion have high numbers of Pf4 in their airway secretions. Given the known severe damage in the airways of *Pa*-infected pwCF, we hypothesized a high Pf4 burden can affect airway healing and inflammatory responses. In the airway, basal epithelial cells (BCs) are a multipotent stem cell population critical to epithelium homeostasis and repair. We sought to investigate the transcriptional responses of BCs under conditions that emulate infection with *Pa* and exposure to high Pf4 burden. Methods: Primary BCs isolated from pwCF and wild-type (WT) donors were cultured in vitro and exposed to Pf4 or bacterial Lipopolysaccharide (LPS) followed by transcriptomic and functional assays. Results: We found that BCs internalized Pf4 and this elicits a strong antiviral response as well as neutrophil chemokine production. Further, we found that BCs that take up Pf4 demonstrate defective migration and proliferation. Conclusions: Our findings are highly suggestive of Pf4 playing a role in the pathogenicity of *Pa* in the airways. These findings provide additional evidence for the ability of inoviruses to interact with mammalian cells and disrupt cell function.

## 1. Introduction

Among the inoviruses, the Pseudomonas Pf4 bacteriophage is capable of infecting its host without causing lysis and infected bacterial cells continue to divide and produce virus indefinitely [1]. The ssDNA phage genome—between 7 and 12 kb long—integrates into the Pa genome as a lysogen, and 0.8–2 um virons are continuously extruded from the bacterial cell envelope without lysis [2,3,4,5].

People with cystic fibrosis (pwCF) are particularly prone to chronic airway infection with Pa. CF is an autosomal recessive genetic disease caused by mutations in the cystic fibrosis transmembrane conductance regulator (CFTR) gene, leading to airway mucus buildup and chronic infections. Most pwCF are chronically infected with Pa by adulthood [6] and this infection becomes a major contributor to disease progression. Chronic Pa infection is associated with declining lung function, frequent episodes of pulmonary exacerbations, and increased mortality [7,8,9]. Pa is particularly pathogenic in pwCF because of its ability to form robust biofilms. These are slimy communities of polymers and microbes that allow Pa to colonize airways and other surfaces and to evade both antibiotics and the host immune response [10,11,12,13].

In addition to bacterial burden, our group and others have shown that a high proportion of Pa-infected pwCF carry strains that harbor Pf4 [4,14,15,16]. We found approximately 40% of pwCF infected with Pa also have a high burden of Pf4 present in their sputum, with 106–1011 virions/mL detected. Other studies have likewise found Pf phages in many Pa strains [4,16,17]. The impact of this high burden of Pf4 in infected airways is not fully understood.

As an inovirus, the Pf4 virion is a tubular structure formed by several thousand units of its major coat protein GP8 around a core of single-stranded circular DNA [18]. The GP8 coat provides the virions with a highly negative surface charge, and large numbers of Pf4 assemble polymers into liquid crystal structures with depletion attraction forces [19,20,21]. This property contributes to the assembly into structures that encases the bacteria and contributes to the persistence of Pa biofilms [20,22]. Pf production is mediated by the excisionase XisF4 and suppressed by the repressor C protein Pf4r, but this is relieved in the setting of Pf superinfection and other cues [23,24].

We have previously also demonstrated that Pf4 engages anti-viral pattern recognition receptors in myeloid cells [25,26]. We have also shown that among pwCF, Pf4 carriage is associated with chronicity of Pa infection, older age, larger decreases in lung function during pulmonary exacerbation, and antibiotic resistance [14]. Other investigators have reported that Pf4 can enhance susceptibility to some antibiotics and reduces Pa virulence [27,28].

Airway basal epithelial cells (BCs) are a stem cell population playing critical roles in homeostasis and the repair of airway epithelium. These cells make up approximately 30% of the pseudostratified epithelium of the tracheobronchial tree and are present throughout the airways, differentiating to regenerate key airway cell types [29,30]. BCs are critical in injury response and the repair of airway tissue [31], and are of interest for their roles in chronic respiratory disease and contribution to inflammation in the airways of pwCF. BCs also play a role in innate immunity in the airway; they possess Toll-like receptors (TLR) for recognition of pathogen-derived molecules and are capable of secreting inflammatory mediators, chemokines, and antimicrobial substances in response to infectious stimuli [32,33]. Previous reports in explanted lung tissue from end-stage lung disease in pwCF indicate that BCs are a heterogenous cell type, with altered phenotypes [34].

Building on previous work establishing Pf4 as a virulence factor produced by Pa and growing evidence for the ability of other inoviruses to interact with mammalian cells in other contexts [35,36,37], we hypothesized that the high Pf4 burden during the course of chronic Pa infection can affect airway healing and inflammatory responses. In this work, we specifically investigated the transcriptional and functional responses of BCs to Pf4 in the context of bacterial stimulation.

## 2. Materials and Methods

### 2.1. Basal Cell Culture

Nasal brushings were acquired after receiving informed consent under a human-subjects-approved protocol (Stanford IRB # 42710) from a CF patient with CFTR mutations 1288insTA and 406-1G>A, a CF patient homozygote for the CFTR mutation F508del, and from a healthy volunteer known to be wild-type at the CFTR locus (WT). Samples were procured and processed as described previously [38,39]. Once confluent, cells were treated for 24 h with Phosphate Buffered Saline (PBS) as a control, 1 × 10^10^ pfu/well of purified Pf4 preparation (an effective MOI of 1000 phage copies per eukaryotic cell), 5 μg/mL LPS (Cat. No. tlrl-eblps, Invivogen, San Diego, CA USA), or their combination. In addition to evaluating responses to Pf4, we included as controls the *E. coli* filamentous phage Fd and the Pa lytic phage DMS3vir. These phages were prepared as described previously [26,40]—in short, PAO1 was spiked with 10^9^ Pf4 pfu/mL and grown overnight at 37 °C. Bacteria were removed by centrifugation at 6000× *g* for 5 min, and supernatant was treated with 1 μg/mL of DNase I (Roche, Cat. No. 4716728001) for 2 h at 37 °C before sterilization by vacuum filtration through a 0.22 μm filter, after which it was precipitated by addition of 0.5 M NaCl and 4% polyethylene glycol (PEG) 8000 (Cat. No. P2139, Millipore Sigma, Burlington, MA, USA). After pelleting by centrifugation at 15,000× *g* for 20 min, a second purification step with PEG 8000 was followed. The purified phages were suspended in sterile PBS and dialyzed in 10 kDa molecular weight cut-off tubing (Cat. No. 88243, ThermoFisher Scientific, Hampton, NH, USA) against PBS and filter-sterilized. The purified phage preparations were quantified via plaque assay and verified as endotoxin-free via Purpald assay [41].

### 2.2. Single-Cell Sequencing

Single-cell suspensions of BCs from 1288insTA/406-1G>A CF and WT donors treated in culture as detailed above were prepared by lifting adherent cells using trypsin digestion. Dead cells were removed using the MACS Dead Cell Removal kit (Miltenyi Biotech, San Jose, CA, USA). Cells were then washed and counted as per manufacturer’s instructions and libraries were prepared using the 10x NEXT GEM 3′ Gene Expression Library scRNA Seq v3.1 reagents and workflow (10x Genomics). Library concentration and quality were confirmed by BioAnalyzer (Agilent, Santa Clara, CA, USA). Libraries were sequenced on the HiSeq PE150 platform (Illumina, San Diego, CA, USA). Quality control of raw data was performed using FASTQC (v 1.0.0), and reads were aligned to the GRCh38-2020 Human Reference Genome using CellRanger (v 7.2.0) to generate counts. An average of 8328 cells were sequenced across all conditions with an average depth of 29,962 reads/cell. Demultiplexed data was analyzed in R using the Seurat 4.0 package [42], and dimensionality reduction and display of data were performed by utilizing Uniform Manifold Approximation and Projection (UMAP) [43]. Marker genes for clusters were characterized using the TopGO R package (release 3.18) [44].

### 2.3. qRT-PCR

BCs from additional WT and homozygote F508del CF donors were cultured and treated as described above, and total RNA was extracted using the Rneasy RNA extraction kit (QIAGEN, Germantown, MD, USA). RNA was used to synthesize cDNA through reverse transcription (Applied Biosystems, Waltham, MA, USA). Expression of genes of interest was quantified by qRT-PCR. Primers sequences were obtained from Primerbank and previously published work, and were as follows: GAPDH [45], OAS1 (Primerbank #74229012c1), OAS2 (Primerbank #74229020c1), IRF7 (Primerbank #98985817c1), and IFIT3 (Primerbank #197276657c1). Three independent validation experiments were performed for CF cells, and two independent validations were performed for WT cells.

### 2.4. Microscopy

For microscopy analysis, BCs were seeded on glass 12 mm #1.5H precision coverslips (Cat. No. CG15CH, Thorlabs, Newton NJ, USA) coated with poly-L-lysine (Cat. No. P8920, Sigma, St. Louis, MO, USA) or 0.2% poly-l-ornithine (Cat. No. P3655, Sigma) according to the manufacturer’s instructions at a density of 1 × 10^6^ cells/well in a 6-well plate. After overnight culture to allow for adhesion, cells were treated for 3 h with either PBS or AF-488 labeled Pf4 at 1 × 10^11^ pfu/mL. Labeling of Pf4 was performed with a TFP ester labeling kit (Cat. A37570, ThermoFisher Scientific) for one hour followed by separation on a 10 × 300 mm gel filtration column. Viability and concentration of the labeled Pf4 was verified by plaque assay, as we have described before [26]. Cells were then fixed in 4% paraformaldehyde, permeabilized in 0.1% Triton X-100, incubated with primary anti-EEA-1 antibody (Cat. MA514794, anti-EEA-1 Rabbit IgG, ThermoFisher Scientific) for 2 h, and incubated with secondary antibody (Cat. A27040, goat anti-rabbit AF-647, ThermoFisher Scientific) for 30 min. Cells were then stained with phalloidin-CF555 (Cat. BT00040, Cambridge Biosciences, Cambridge, UK) for 20 min, followed by DAPI (Cat. D21490, Invitrogen, Waltham, MA, USA) for 10 min. Specimens were imaged with a confocal system (DMX BLAZE 3D SIM, GE Healthcare, Chicago, IL, USA). Images were acquired using the Leica Application Suite X (Leica Microsystems, Wetzlar, Germany) and processed in ImageJ.

### 2.5. ELISA

Supernatants from BC cultures were assessed for cytokine content as per manufacturer’s instructions. CXCL1 (R&D systems, Minneapolis MN, USA), IL-8/CXCL8 kits (R&D systems), CXCL5 (Biolegend, San Diego, CA, USA), and CXCL10 (Biolegend) kits were used.

### 2.6. Proliferation/Migration Assay

BCs from CF donor 1288insA/406-1G>A and the WT donor were plated onto collagen-coated 96-well plates and once actively proliferating were treated with PBS control, 10^10^ pfu/mL Pf4, 1 μg/mL LPS, or their combinations. The plate was then placed on an Incucyte incubator (Sartorius Lab Instruments, Goettingen, Germany) at 37 °C and 5% CO_2_ for time-lapse phase-contrast imaging over a 48 h time span. Conditions were run in quadruplicate, and progression to confluence was quantified by image analysis as we have described before [46].

## 3. Results

### 3.1. WT and CF BCs Cluster into Functionally Distinct Subsets

**Figure 1:**
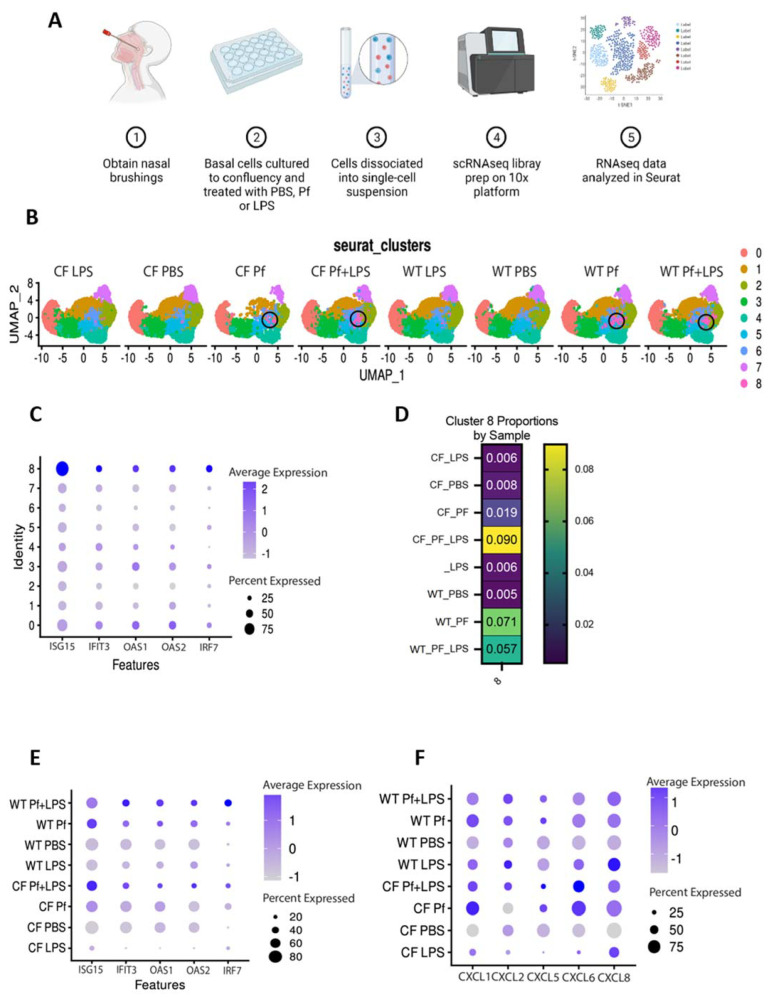
Single cell RNA sequencing of WT and CF BCs demonstrate distinct responses to Pf4. (**A**) Diagram of experimental workflow. (**B**) UMAP plots for all experimental conditions (Pf4, LPS and/or PBS control) for CF and WT cells identify specific functional clusters. Among cells exposed to Pf with or without LPS a cluster with an antiviral gene expression signature was clearly apparent (Cluster 8, circled) (**C**) Dot plot showing higher average expression of ISG15, IFIT3, OAS1, OAS2, and IRF7 by cluster identity. (**D**) Proportion of cells in cluster 8 by experimental condition. (**E**) Dot plot showing average expression of markers ISG15, IFIT3, OAS1, OAS2, and IRF7 across samples. (**F**) Dot plot showing average expression of markers CXCL1, CXCL2, CXCL5, CXCL6, and CXCL8 across all samples. Graphical schematics created with BioRender.com.

A schematic of the workflow followed for single-cell RNA sequencing of treated BCs from the WT donor and CF donor 1288insTA/406-1G>A is shown in Figure 1A. Sequence data were subjected to unsupervised clustering and visualized by UMAP. Unsupervised clustering was used to identify transcriptionally distinct subsets of BCs without relying on manual annotation restricted by current knowledge of BC heterogeneity. Under untreated control conditions, comparing CF and WT BCs, we identified seven clusters as assessed by Gene Ontology (GO) term enrichment for cluster-defining genes (Figure S1C). CF BCs had fewer cells in Cluster 0, functionally defined via GO analysis as being involved in ribosome biogenesis and regulation of apoptosis, Cluster 1, defined by genes corresponding to cytokine-mediated signaling as well as neutrophil degranulation and chemotaxis, and Cluster 3, defined by genes involved in epidermal and keratinocyte cell differentiation. Conversely, CF BCs were enriched for Clusters 4, defined by positive regulation of proliferation and leukocyte chemotaxis, and 6, defined by genes involved in DNA metabolic processes and mitosis.

Analysis of the gene expression profiles in response to our experimental treatment conditions identified nine clusters (Figure 1B). Both CF and WT cells treated with Pf4, whether with or without LPS, contained a small but significant cluster defined by strong upregulation of antiviral response genes and interferons, including ISG15, IFIT3, OAS1, OAS2, and IRF7 (Figure 1B–E). A set of chemokines was likewise differentially expressed in the setting of Pf4 exposure (Figure 1F). Together, these data demonstrate that distinct patterns of gene expression, consistent with anti-viral responses, were observed in the setting of Pf4 exposure.

**Figure 2:**
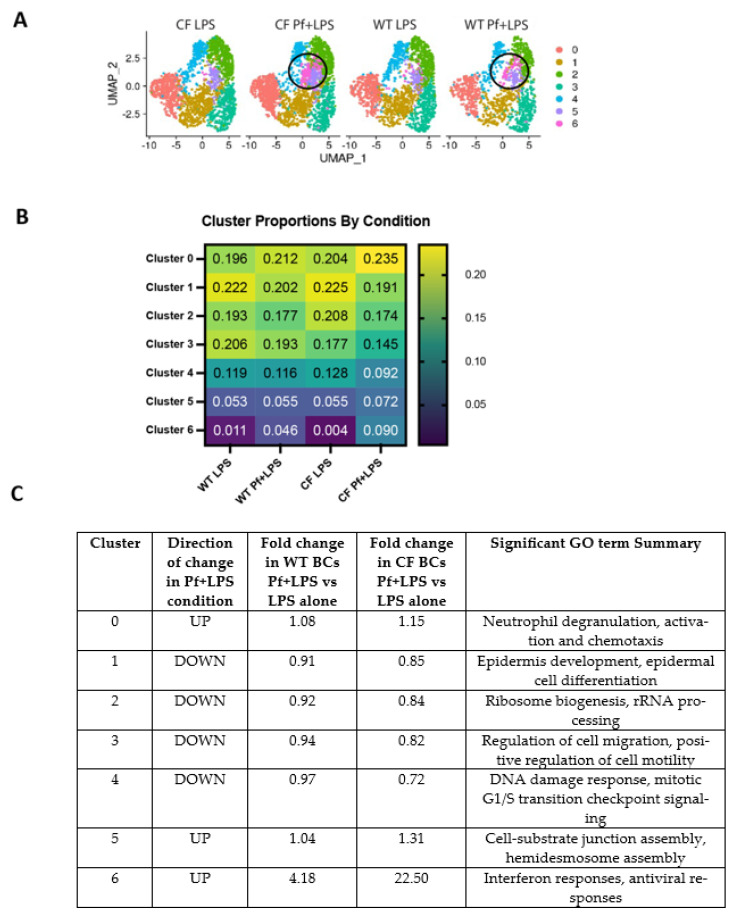
WT and CF cells exhibit differing responses between Pf4 and Pf4 + LPS experimental exposure conditions. (**A**) UMAP of WT and CF BCs comparing only cells stimulated with Pf ± LPS. Cluster corresponding to antiviral responses circled in black. (**B**) Proportion of cells in defined clusters for WT and CF cells between LPS and Pf + LPS conditions. (**C**) GO terms corresponding to each cluster across categories.

To better distinguish the specific responses that will be elicited by Pf4 itself in the airway, we further analyzed the data blocking only for the Pf4 +/− LPS since this models a comparison of the responses of BCs to Pf4 in the presence of Pa versus just by itself (Figure 2). Cluster 0 is defined by GO terms related to neutrophil activation and chemotaxis, indicating a cell population which primarily secretes neutrophil chemoattractants. GO term enrichment identified six distinct clusters: Cluster 1 is defined by GO terms related to epidermal development and differentiation and likely consists of differentiating cells. Cluster 2 is defined by ribosome biogenesis and rRNA processing, indicating this cell grouping is likely undergoing differentiation as well—it is well-established that stem cells maintain low translation rates independently of cell cycle state and dramatically increase ribosome biogenesis during differentiation [47]. Cluster 3 is defined by GO terms relating to positive regulation of cell motility and migration. Three additional minor clusters were defined by G1/S cell cycle transitioning (Cluster 4), cell–substrate junction/hemidesmosome assembly (Cluster 5), and, in the case of the smallest cluster, antiviral responses (Cluster 6). Overall, both WT and CF BCs showed a clear response to LPS stimulation with an enrichment of inflammatory response genes (Figure S2A). Interestingly, the most significant gene sets enriched in CF LPS-treated cells as compared to WT LPS-treated cells were all related to unfolded protein responses, indicating that although the largest difference in these cells remains the presence of misfolded CFTR expected from the presence of mutations, the CF and WT cells have a common LPS response (Figure S2B). The proportion of cells corresponding to the antiviral cluster represented 4.6% of cells in the WT BCs Pf + LPS condition, and this was significantly larger in the CF Pf + LPS condition at 9.0% of cells (Figure 2B). This represented increases from the LPS alone conditions of ~4-fold for WT cells, and ~20-fold for CF cells (Figure 2B,C).

Apoptotic markers were not increased in the Pf + LPS conditions and did not correlate with the antiviral cluster marker genes IFI6 or ISG15 (Figure S3A,B), and feature plots of immune cell markers show little to no cells contaminating these samples (Figure S4). Together, these data indicate that this antiviral signature is due to a direct response of BCs to Pf4 and not driven by contaminating or apoptotic cells.

**Figure 3:**
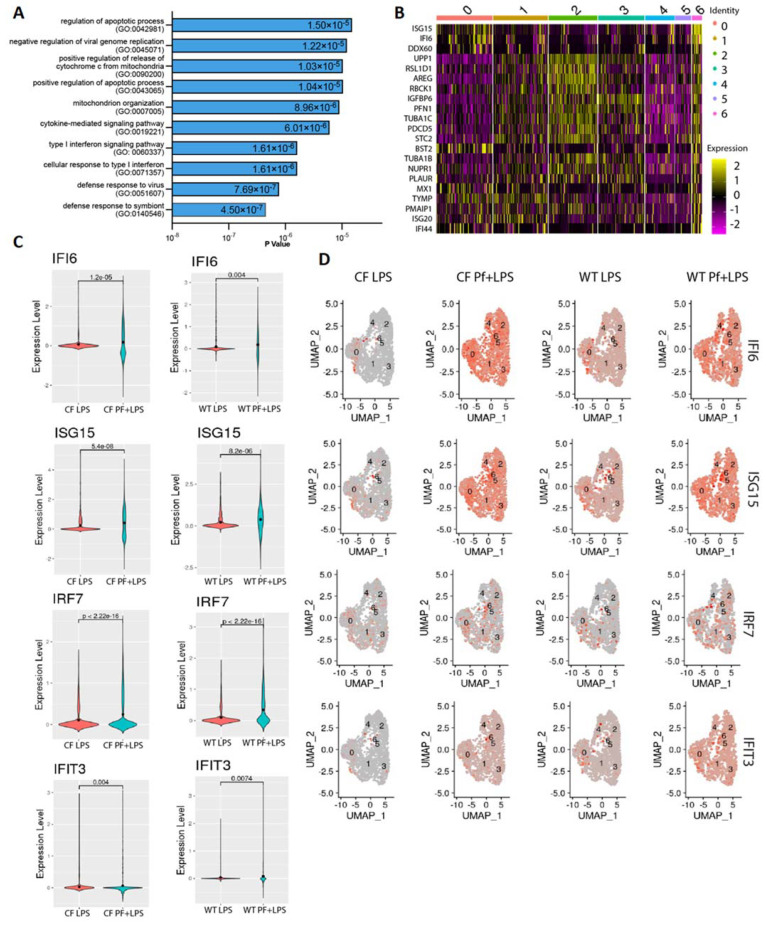
BCs initiate antiviral responses to Pf4 phage. (**A**) GO term enrichment for top upregulated genes in antiviral cluster. (**B**) Gene expression pattern across all clusters for top upregulated genes of antiviral cluster. (**C**) Gene expression for individual antiviral genes across control and Pf-stimulated conditions cystic fibrotic (left) and WT (right) cells. (**D**) Overall expression of individual antiviral genes in WT and CF cells exposed to LPS or Pf + LPS.

Apart from the antiviral cluster, several other clusters showed changes in cell proportion between conditions. Specifically, following Pf + LPS treatment, both donors had lower proportions of cells in clusters related to epidermal differentiation, regulation of cell migration, and DNA damage response/mitotic cell cycle transitioning (Figure 2C, Figure 3B–D and Figure S5).

These findings indicate that the presence of Pf4 appears to hinder or divert BCs from homeostatic and infection/injury-repair processes. Notably, CF cells had larger decreases in these clusters than WT cells. In addition, both WT and CF also showed an increase in the cluster defined by neutrophil activation/chemotaxis, suggesting that the presence of Pf4 increases the number of cells expressing chemotactic factors as compared to LPS stimulation alone.

### 3.2. Pf4 Induces an Antiviral Response in a Subset of BCs

In order to further characterize the Pf4-driven antiviral cluster, we identified the top-expressed individual genes unique to this subgroup of cells. These sets of genes were virtually identical between WT and CF, and included such canonical type I interferon-driven genes as DDX-, OAS-, and ISG-family genes (Table S1, Figure 3B). Top GO terms for this gene set included cellular response to type I interferon, negative regulation of viral genome replication, and cellular response to dsRNA (Figure 3A). This last enrichment was particularly interesting given our previous findings that Pf4 phage triggers TLR3, a dsRNA sensor [26]. Furthermore, although the antiviral cluster exhibited the highest expression of antiviral genes, several of these genes were also expressed to varying degrees across all clusters. Notably, overall expression of the top-enriched antiviral genes was higher in the Pf4 + LPS condition as compared to the LPS-only condition for both donors (Figure 3C,D). These antiviral genes were shown to be upregulated in Pf4 only condition as well, indicating the signature is independent of LPS stimulus (Figure 1E).

**Figure 4:**
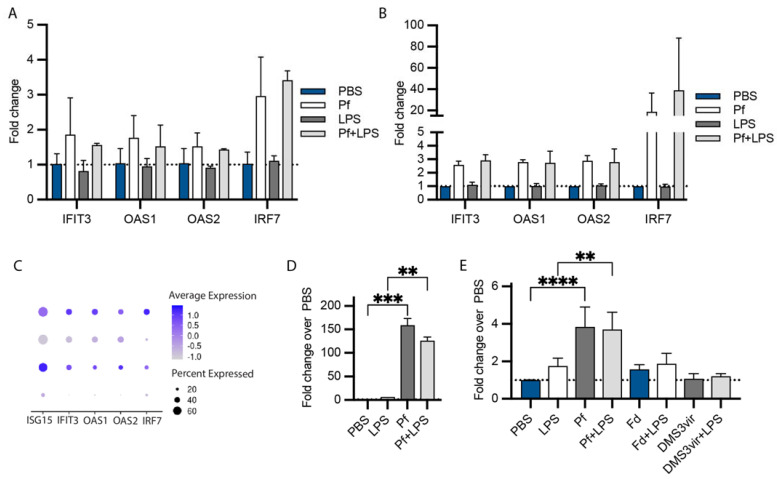
Basal cells from additional donors validate antiviral signature in response to Pf4 phage. RT-qPCR of WT (**A**) and CF (**B**) basal cells incubated with Pf4 10^10^ pfu/mL ± LPS 5 ug/mL for 24 h. (**C**) Expression of the selected validation genes in sequencing dataset. Protein quantification of CXCL10 in supernatants from WT (**D**) and CF (**E**) basal cells stimulated as in (**A**) as determined by ELISA, with 10^10^ pfu/mL of additional phage controls. Significance between different conditions (** *p* < 0.01, *** *p* < 0.001, **** *p* < 0.0001) was assessed by ANOVA followed by Holm-Šídák test for multiple comparisons.

We validated the upregulation of these type I interferon response genes in BCs from an additional WT donor and additional CF donor homozygote for F508del by qRT-PCR (Figure 4). We chose validation targets with a range of expression intensities, both top-expressed and lower-expression genes (Figure 4C). We found that, as expected, exposure of BCs to Pf4 both with and without additional LPS stimulation induced upregulation of the antiviral genes IFIT3, OAS1, OAS2, and IRF7 at levels equal to or above LPS-only gene induction (Figure 4A,B). In particular, IRF7 was exclusively induced by Pf4 phage stimulation. CF cells showed a greater induction of antiviral genes than WT cells in response to Pf4, with IRF7 in particular being much more highly expressed.

We also evaluated BCs production of the canonical antiviral response chemokine CXCL10. We found that both WT and CF cells produce much higher amounts of CXCL10 in response to Pf4 stimulation as compared to LPS (Figure 4D–E). Notably, our control phages Fd (produced in *E. coli*) and DMS3vir (produced in Pa) did not induce significant CXCL10 secretion in BCs (Figure 4E).

We confirmed that induction of Pf4-driven antiviral responses could not be driven by LPS or free nucleic acid contamination in our Pf4 phage preparations. The LPS content of our Pf4 phage reagent was ~25 ng/mL at the dilution used, much lower than the 5 µg/mL of LPS used as a stimulus. Effective LPS contents of Fd and DMS3vir phages at the dilutions used were 2 μg/mL and 20 ng/mL, respectively, and these phages did not induce antiviral chemokine production. Extensive nuclease digestion was also performed on our phage preparations in order to eliminate free DNA or RNA.

**Figure 5:**
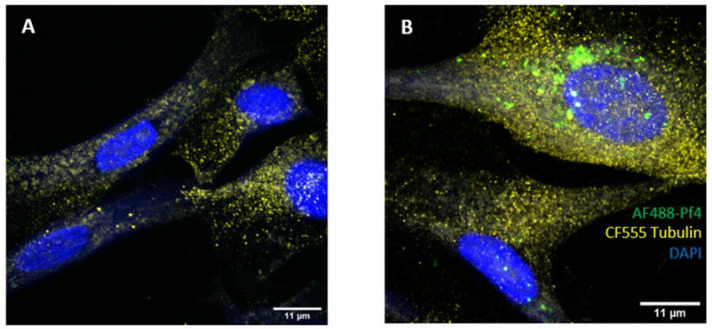

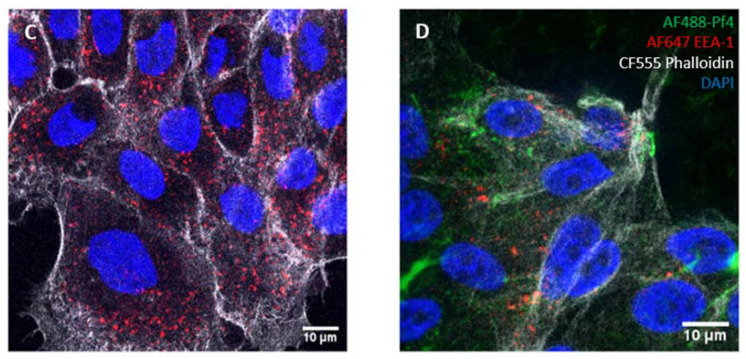
CF BCs internalize Pf4 phage. (**A**) Untreated BCs or (**B**) labelled Pf4 treated BCs stained with tubulin-CF555 demonstrate uptake of Pf4. To further determine intracellular localization, (**C**) Untreated BCs or (**D**) labelled Pf4 treated BCs stained with endosomal marker EEA-1-AF647 demonstrate Pf4 localization with endosomes. Confocal images taken at 100× magnification.

Finally, given that our findings implicate a role for TLR3—an endosomal sensor—in sensing Pf4 phage, we investigated whether BCs were capable of internalizing phage. Punctate Pf4 phage uptake was clearly observed in BCs exposed to Pf4 for 24 h, often overlapping with EEA-1 staining, an early endosome marker, indicating internalization of phage particles (Figure 5).

### 3.3. Pf4 Phage Increases Neutrophil Chemoattractant Production in BCs

**Figure 6:**
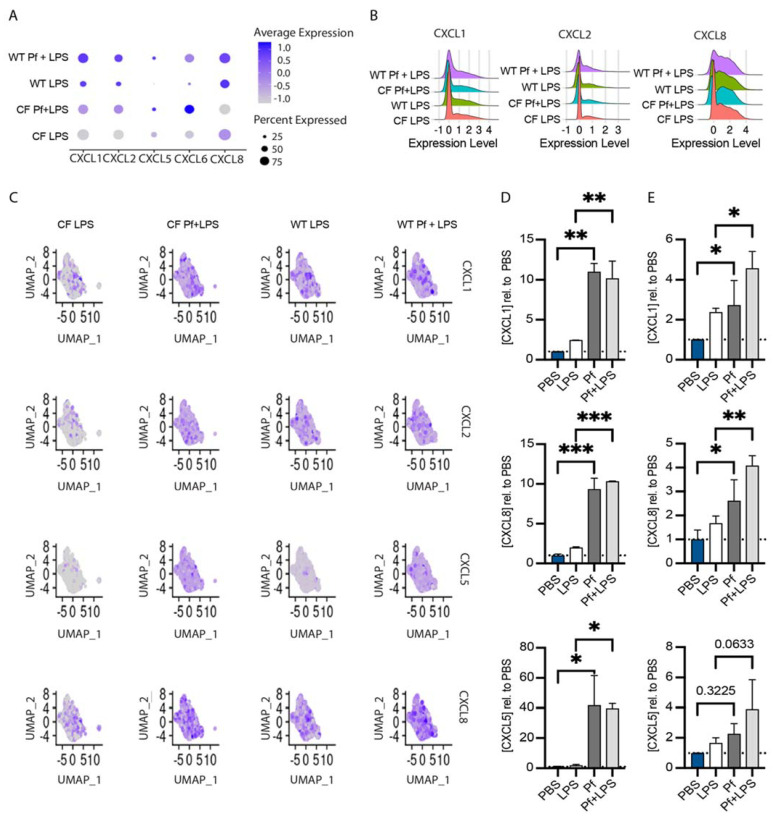
Pf4 induces production of neutrophil chemotactic factors in WT and CF basal cells. Expression (**A**) and ridge plots (**B**) of selected neutrophil chemoattractant genes in cluster 0 of the 10x sequencing dataset. (**C**) Feature plots showing neutrophil chemokine expression across basal cell clusters. Protein concentration of neutrophil chemoattractants in supernatants from WT (**D**) and CF (**E**) basal cells incubated with 5 ug/mL LPS ± 10^10^ pfu/mL Pf4 phage for 24 h as determined by ELISA. Significance between different conditions (* *p* < 0.05, ** *p* < 0.01, *** *p* < 0.001) relative to PBS control (dotted line) was assessed by ANOVA followed by Holm-Šídák test for multiple comparisons.

We also chose to further investigate Pf4-mediated effects on basal cell chemokine expression. A cluster defined by neutrophil activation and chemotaxis gene upregulation (Cluster 0, Figure 2) increased by 8–15% with Pf4 and LPS stimulation as opposed to LPS-only treated cells in both WT and CF BCs cultures (Figure 6A). This subset of cells expressed genes such as CXCL1, CXCL2, and IL-8/CXCL8 (Figure 6B), all potent neutrophil migration and activation factors. We evaluated protein production of a subset of these factors in BCs cultures from additional WT and CF donors as described above and confirmed that Pf4 stimulation strongly increases production of CXCL1, CXCL8, and CXCL5 above the level induced by LPS alone in both donors (Figure 6D), though the magnitude of response was stronger in WT cells. These markers were also found to be upregulated in Pf4 only conditions, indicating this effect is independent of LPS stimulus (Figure 1E).

### 3.4. Pf4 Impairs the Ability of BCs to Reach Confluence

Given our finding of BCs internalizing Pf4, we asked the question if this could impact the epithelial regeneration capabilities of infected BCs. We investigated then for an effect on BC proliferation and migration, key processes for effective repair functions. We noted that a subset of BCs in both the WT and CF datasets (Figure 2C, Cluster 3) expressed cellular migration and motility-related genes. This cluster consisted of ~20% of all basal cells, and was decreased by 6% and 18%, respectively, in WT and CF cells exposed to Pf4 phage (Figure 2B,C). Although there were no significant differences in cell cycle state between conditions in cells of either genotype in our single-cell dataset, this dataset consisted of confluent cells which had moved past active proliferation and migration. Therefore, in order to further understand the effects of Pf4 on BC proliferation towards confluence, we tracked cellular growth in sparsely seeded, actively proliferating BCs over the course of 48 h.

**Figure 7:**
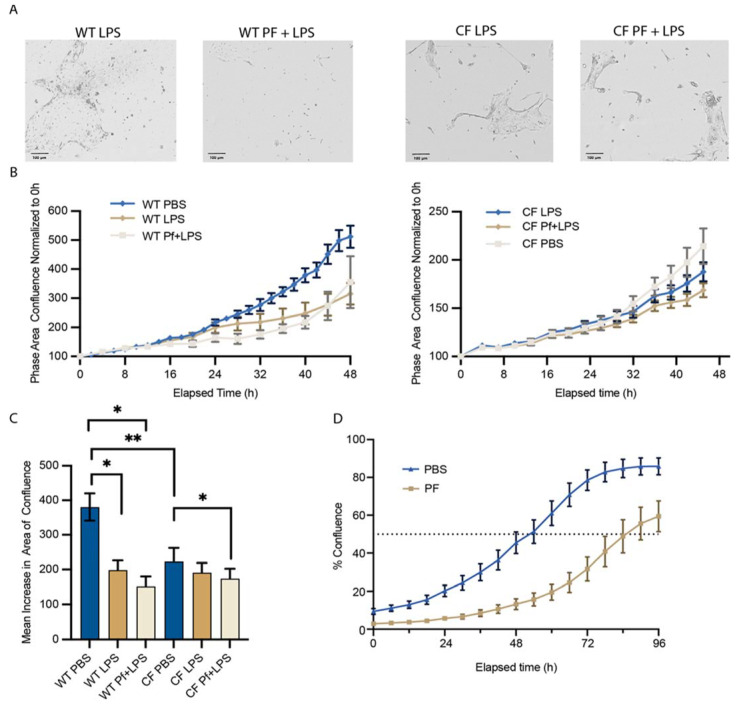
Pf4 decreases BCs progression towards confluence. (**A**) Representative 200× images of WT cells treated with LPS (**left**) and Pf + LPS (**right**) shown at 48 h. CF cells treated with LPS (**left**) and Pf + LPS (**right**) shown at 48 h. (**B**) Progression towards confluence shown for WT and CF cells treated with Pf4 and control conditions. (**C**) Mean area increase in confluence at 48 h for both genotypes and all conditions shown. Means and SD estimated by generalized linear model (GLM) and comparisons between different groups assessed by Tukey’s studentized range test (* *p* < 0.05, ** *p* < 0.01). (**D**) Time to 50% confluence (dotted line) of the seeded area for WT cells in the presence of Pf4 or PBS control was assessed between 54 and 86 h (*p* = 0.012).

The exposure of BCs to Pf4 appeared to delay progression towards confluence, with a stronger phenotype in CF cells as compared to WT cells (Figure 7A,B). After 48 h, untreated CF cells had significantly lower confluence that WT cells (Figure 7C). WT cells exposed to Pf4 and LPS had a substantial decrease compared to control conditions, with no significant difference between Pf4 and LPS treatment alone but with their combination inducing the most pronounced significant effect (Figure 7C). CF cells treated with Pf4 and LPS had the lowest confluence compared to control conditions (Figure 7C).

These data are in line with the greater magnitude of Cluster 3 depletion in CF cells versus WT cells with Pf4 treatment. To further delineate the effect of PF4 by itself on the kinetics of confluence, we extended the period of observation to 96 h with a second set of WT cells treated with either PBS control or Pf4. We observed that control WT cells reached 50% confluence by 54 h, as opposed to Pf4-treated cells that took 86 h to reach that level of confluence (Figure 7D).

## 4. Discussion

In our work here, we show that the inovirus Pf4 is recognized as a virus by BCs, is internalized by these cells, and shifts the transcriptional response to LPS, as well as affecting essential cell functions. With this, we demonstrate for the first time, to our knowledge, the ability of an inovirus to invade airway stem cells and induce a number of transcriptional and phenotypic changes. Although lytic phages used in the context of phage therapy have been shown to enter cells, this occurs in mature cells on the mucosal surface and is hypothesized to occur in association with its bacterial host [48]. Further, both WT and engineered phages have also been shown to induce shifts in transcriptional programs in mammalian cells [35,49]. Here, we are showing this to occur to BCs, which has important implications for tissue regeneration, as our proliferation data demonstrates. Since Pf4 is actively extruded by Pa and is highly abundant in the lungs of some pwCF, we believe that this finding identifies a novel and previously unsuspected mechanism involved in the pathogenicity of chronic Pa infections in the CF airway.

BCs are a key member of the airway ecosystem, regenerating multiple cell types in response to injury and infection [30]. We first show that BCs from both WT and CF donors exposed to LPS to model bacterial infection conditions exhibit transcriptional heterogeneity and segregate into functionally distinct clusters, falling into four major groups each comprising about 20% of cells in the population (Figure 2C).

Our previous work suggests that Pf4 is internalized by multiple immune cell types and induces antiviral responses [26], and further work in a cohort of pw CF indicated that Pf4 is associated with worsened clinical outcomes [14]. Building on this work, we find here that airway BCs also internalize and mount antiviral immune responses to Pf4. The antiviral gene cluster increases by 4- and 20-fold, respectively, in WT and CF cell populations. In addition, overall expression of antiviral genes such as IFI6, DDX60, and ISG1 is increased overall in both WT and CF cells. Although little work exists on antiviral responses in BCs, it is known that this cell type can be infected by viruses such as rhinovirus and respiratory syncytial virus, and that TLR3 is a major mediator of the airway epithelial cell response to these pathogens [50,51,52]. We propose that BCs sense Pf4 through TLR3 and activate an antiviral transcriptional program. BCs express TLR3 mRNA, rendering support to this potential mechanism. Furthermore, we show that Pf4, but not LPS alone, specifically induces expression of IRF7, a transcription factor downstream of TLR3/TRIF signaling primarily induced by type I interferons and viral sensing [53].

We also find that a large subpopulation of BCs are characterized by increases in neutrophil-activating gene expression upon Pf4 exposure. We find that these responses are not prompted by other bacteriophages used here, suggesting this effect is specific to Pf4 and not a common characteristic of phage virion exposure. This finding is in line with previous work on viral sensing and neutrophil recruitment: all major neutrophil chemokines are produced during respiratory viral infection, and neutrophil influx to the site of infection is robust and sustained [54]. In particular, TLR3 deficiency has been shown to decrease neutrophil recruitment to the lung, underscoring the involvement of this viral sensor in chemokine production [55]. While Pf4-exposed BCs are producing antiviral factors and increasing chemokine production, other cellular functions are neglected. The proportion of cells in Clusters 1–4—corresponding to differentiation, replication, and cell migration—decreased by approximately 5–10% for WT Pf-stimulated cells, and 20–40% for CF cells. As a functional correlate of this finding, we show that Pf4 exposure decreases the proliferation/migration rate of both WT and CF BCs. This finding is in line with previous reports of [56] phage interfering with cell migration and proliferation [57], but with the contrasting fact in our work that Pf4 is produced by Pa, indicating its use by Pa as an additional pathogenicity tool at its disposal.

Taken together, these findings suggest that the presence of this inovirus, known to be present in high concentrations (up to 10^11^ copies/mL) in the airway of pwCF chronically infected with Pa, significantly alters the BC response to bacterial stimulation. Notably, increased expression of neutrophil activation and chemoattraction factors provides evidence for the BC population actively participating in the recruitment of neutrophils to the CF airway. Hyperaccumulation and dysfunction of neutrophils is a known major contributor to airway damage and chronicity of infection in people with CF [58,59,60]. In pwCF chronically infected with Pf4-producing *P. aeruginosa*, our results suggest that neutrophil burden would be higher than in the absence of Pf4, leading to worse patient outcomes in line with our previous work [14]. Perhaps of greatest importance, widespread antiviral signaling by BCs would be expected to promote an aberrant response to Pa infection. We will expect that the presence of bacterial biofilm will induce an immune response, though this will not be expected to present with the antiviral signature that we find here. Clinically, co-infection with both a viral and bacterial pathogen has been reported to impair antibacterial immune responses: viral infection is known to increase susceptibility to bacterial colonization and decrease control of bacterial replication in mammalian cells [61,62,63,64]. Finally, impaired BC migration and proliferation in the presence of high Pf4 burden would hinder airway repair and regeneration in the face of chronic bacterial infection. Disrupted healing responses are a known feature of chronic Pa infection in CF and active production of Pf4 might be a mechanism behind this.

Although our work identifies a unique and intriguing aspect of Pa infection pathobiology in the CF airway, there are limitations to recognize. First, we worked exclusively with nasal biopsies as opposed to bronchial airway cells. While these cells originate from the upper airway, it is well established that Pa infection starts in the sinonasal cavity and the nasal epithelium mirrors the pathology and inflammatory response seen in the lower airway [65,66]. Second, our experiments were limited to individuals with severe CFTR mutations. The effect on those with mild mutations or pwCF receiving CFTR modulators is unclear, and ongoing work is focused on these important aspects. Additionally, while the direction of trends was consistent in WT and CF cells, the magnitude of responses to LPS (Figure 3D and Figure 6C) and Pf4 (Figure 4D,E) are more pronounced in cells from WT donors. This is potentially due to slower growth of cells from CF donors (Figure 7B). Further limitations are inherent to the study of mammalian-phage immunology: bacteriophage preparations often contain trace amounts of endotoxin and purification is critical for separating direct versus co-stimulatory effects of phages. Further work is additionally needed for elucidation of what characteristics of Pf4 prompt these responses, as other phages which share either morphology or bacterial hosts failed to generate the same responses. Bacteriophages are an immensely diverse group of viruses, and even within phages infecting *Pseudomonas aeruginosa*, much work remains in understanding these interactions and the scale at which they occur in the human body.

The findings reported here open several new avenues of investigation. Our focus in this work was on BCs, but research into the effects of inoviruses like Pf4 on fully differentiated airway epithelium would be of great interest. Others have reported that viral infection of BCs shifts the differentiation trajectory, raising the question of whether chronic Pf4 exposure would have a similar effect [51]. Furthermore, our work was largely limited to a single highly abundant inovirus previously known to induce antiviral responses. However, given the resurgence of interest in phage therapy to treat highly antibiotic resistant bacteria in the CF airway and other chronic infection settings, the data presented here suggest that we should not think of bacteriophages as inert, non-immunostimulatory particles. Mammalian cell response to bacteriophages remains an important area of further investigation to identify and reduce unintentional off-target effects in phage therapy.

## Data Availability

Raw sequencing data is available in the GEO data repository under accession number GSE200342—“Gene expression profile at single cell level of Basal Cells (BCs) from nasal scrapings of patients with and without cystic fibrosis”.

## Supplementary Materials

The following supporting information can be downloaded at: https://www.mdpi.com/article/10.3390/v16010165/s1, Table S1: Top enriched genes in the antiviral cluster of BCs. Figure S1: Under control conditions WT and CF BCs cluster into functionally distinct subsets. Figure S2: WT and CF BCs exhibit inflammatory responses to LPS; Figure S3: Feature plots of apoptotic markers in BCs; Figure S4: Feature plots of lymphocyte markers in WT and CF BCs; Figure S5: GO analysis of additional clusters from WT and CF BCs.
