# Supplementary material for: The Inovirus Pf4 Triggers Antiviral Responses and Disrupts the Proliferation of Airway Basal Epithelial Cells
Source: Viruses. 2024 Jan 22;16(1):165. doi: 10.3390/v16010165 (PMC10818373; doi:10.3390/v16010165)
Supplement: Supplementary file 1 [file viruses-16-00165-s001.zip › viruses-2829192-supplementary.pdf]

Supplementary Material to article:

"The Inovirus Pf4 Triggers Antiviral Responses and Disrupts the Proliferation of Airway Basal Epithelial Cells"

Table S1: Top enriched genes in the antiviral cluster of BCs.

Figure S1: Under control conditions WT and CF BCs cluster into functionally distinct subsets.

Figure S2: WT and CF BCs exhibit inflammatory responses to LPS.

Figure S3: Feature plots of apoptotic markers in BCs

Figure S4: Feature plots of lymphocyte markers in WT and CF BCs

Figure S5: GO analysis of additional clusters from WT and CF BCs.

Table S1: Top enriched genes in the antiviral cluster. *Values below ~2.23E-308 are represented as 0.

Gene	P-value*	Avg. log2(fold change)	Adj. p-value*
**ISG15**	0	4.04	0
**CCL5**	0	1.61	0
**OASL**	0	3.59	0
**DDX58**	0	2.20	0
**IFIT1**	0	3.12	0
**RSAD2**	0	2.47	0
**IFIT2**	0	3.38	0
**HERC5**	0	2.04	0
**IFIH1**	0	1.88	0
**DDX60**	0	2.36	0
**IFIT3**	0	3.18	0
**IFI44**	0	1.83	0
**ZC3HAV1**	0	2.24	0
**USP18**	0	1.21	0
**ISG20**	0	2.03	0
**CXCL11**	0	2.46	0
**SAMD9**	0	2.20	0
**OAS3**	0	1.19	0
**MX1**	0	2.17	0
**OAS1**	0	1.58	0
**PMAIP1**	0	2.55	0
**OAS2**	0	1.01	0
**NUPR1**	0	2.42	0
**PLAUR**	0	1.94	0
**IFI6**	0	1.70	0
**IFITM3**	0	1.22	0
**PARP14**	0	1.46	0
**IRF7**	0	1.11	0
**EIF2AK2**	0	1.17	0
**GBP1**	0	1.01	0

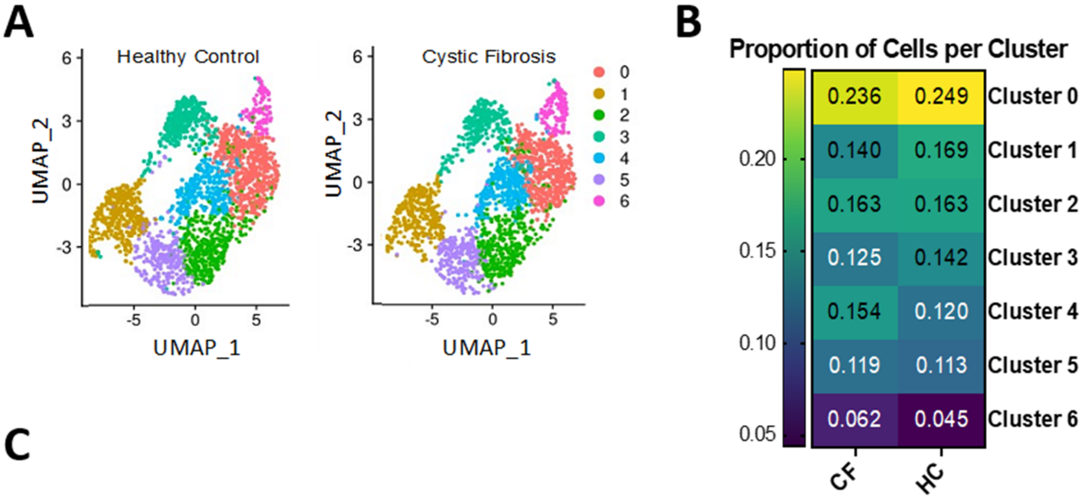

C

Cluster Gene Ontology Markers	
0	Ribosome Biogenesis, rRNA metabolic process, cell-substrate junction assembly, regulation of apoptotic process, regulation of NOS activity
1	Neutrophil degranulation, neutrophil activation, cytokine-mediated signaling pathway, innate immune response, O-glycan processing, neutrophil chemotaxis
2	NADH dehydrogenase activity
3	Keratinocyte differentiation, epidermal cell differentiation, negative regulation of proteolysis
4	Positive regulation of leukocyte chemotaxis, positive regulation of cell population proliferation, positive regulation of angiogenesis
5	Negative regulation of fatty acid metabolic process, positive regulation of stem cell differentiation, negative regulation of immune system process, cellular response to epidermal growth factor stimulus
6	Mitotic spindle organization, microtubule cytoskeleton organization involved in mitosis, mitotic nuclear division, DNA metabolic process

Figure S1. Under control conditions, WT and CF BCs cluster into functionally distinct subsets. (**A**) Unsupervised clustering revealed 7 functionally distinct clusters populated by both WT and CF cells, (**B**) Proportions of cells in clusters vary between CF and WT BCs, (**C**) Gene Ontology enrichment of marker genes reveal functionally distinct gene signatures of clusters.

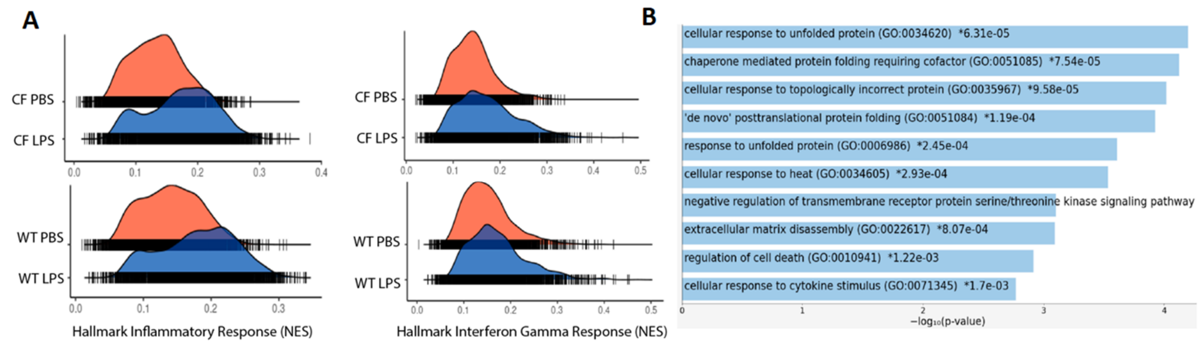

Figure S2. WT and CF BCs exhibit inflammatory responses to LPS. (**A**) GSEA of WT and CF BCs transcriptional response to LPS stimulation. (**B**) GO enrichment of all significantly differentially expressed genes in CF versus WT responses to LPS.

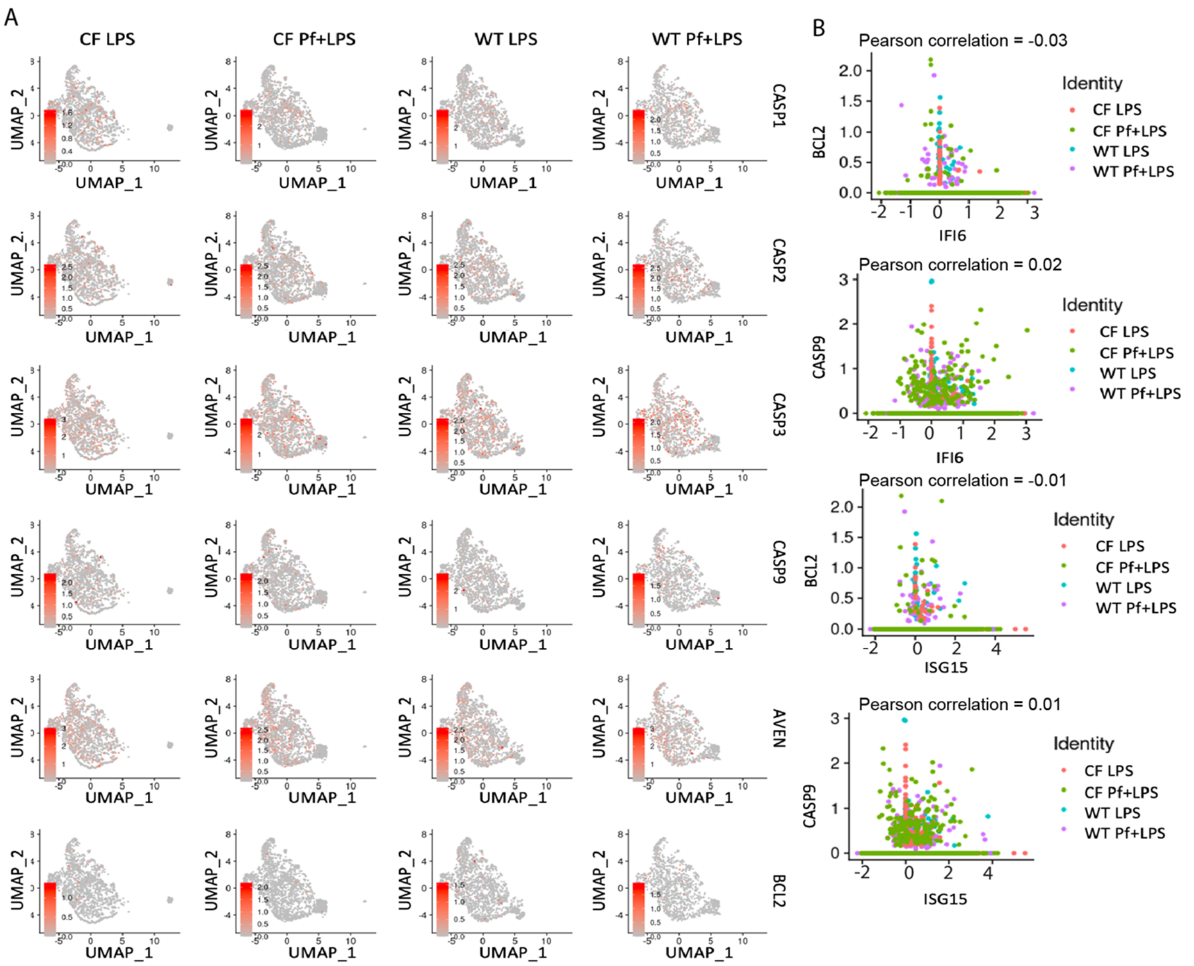

Figure S3. Feature plots of apoptotic markers in BCs. (**A**) UMAP plots for apoptosis markers CASP1, CASP2, CASP3, CASP9, AVEN, and BCL2. (**B**) Feature Scatter plots for BCL2 and CASP9 versus antiviral signature genes ISG15 and IFI6, with Pearson correlation values above plot.

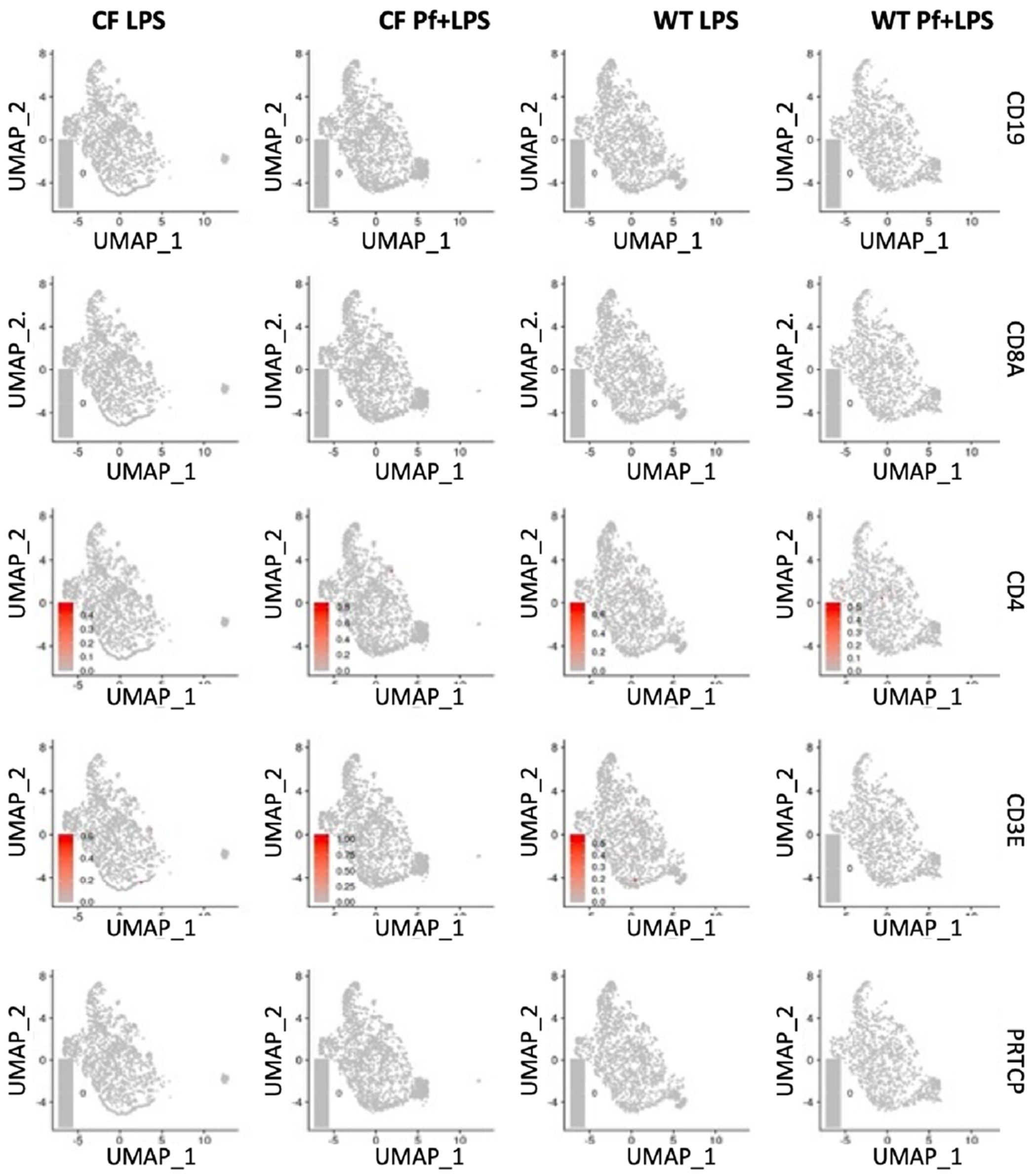

Figure S4. Feature plots of lymphocyte markers in WT and CF BCs. UMAP plots for immune cell markers CD19, CD8A, CD4, CD3E, and PTPRC (CD45) in WT and CF donor cells.

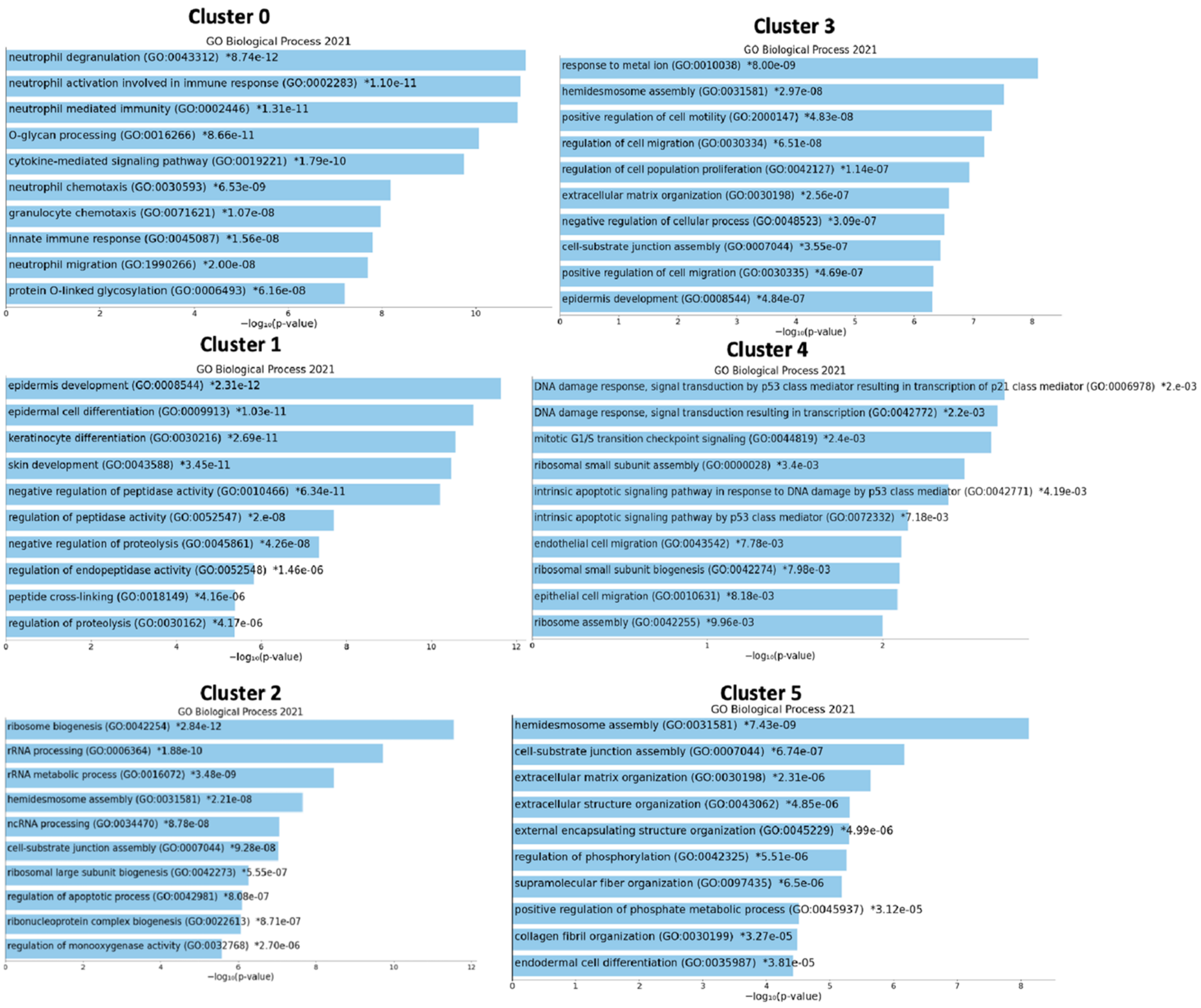

Figure S5. GO analysis of additional clusters from WT and CF BCs. GO term enrichment for top upregulated genes in clusters 0-5 for WT and CF donor cells.
